# Relationship between blood concentration of zinc and selenium and prognosis in post-acute myocardial infarction: A protocol for systematic review and meta-analysis

**DOI:** 10.1371/journal.pone.0333264

**Published:** 2025-10-07

**Authors:** Ana Francisca Teixeira Gomes, Lisandra Mikaely Barboza da Silva, Raissa Beatriz Silvestre Carneiro, Raquel Costa Silva Dantas-Komatsu, Gidyenne Christine Bandeira Silva de Medeiros, Vivian Nogueira Silbiger, Bruna Zavarize Reis, André Ducati Luchessi

**Affiliations:** 1 Postgraduate Program in Health Sciences, Center for Health Sciences, Federal University of Rio Grande do Norte, Natal, Rio Grande do Norte, Brazil; 2 Postgraduate Program in Nutrition, Center for Health Sciences, Federal University of Rio Grande do Norte, Natal, Rio Grande do Norte, Brazil; 3 Multidisciplinary Residency Training Program, Onofre Lopes University Hospital, Federal University of Rio Grande do Norte, Natal, Rio Grande do Norte, Brazil; 4 Postgraduate Program in Pharmaceutical Sciences, Center for Health Sciences, Federal University of Rio Grande do Norte, Natal, Rio Grande do Norte, Brazil; 5 Postgraduate Program in Public Health, Center for Health Sciences, Federal University of Rio Grande do Norte, Natal, Rio Grande do Norte, Brazil; 6 Department of Nutrition, Center for Health Sciences, Federal University of Rio Grande do Norte, Natal, Rio Grande do Norte, Brazil; 7 Translational Medicine, The Hospital for Sick Children, Toronto, Ontario, Canada; 8 Department of Clinical and Toxicological Analysis, Federal University of Rio Grande do Norte, Natal, Rio Grande do Norte, Brazil; University of Messina, ITALY

## Abstract

Acute Myocardial Infarction (AMI) is characterized by the presence of injury caused by an ischemic event, which leads to various complications, including Heart Failure (HF), the most severe functional stage of the heart, reducing both quality of life and life expectancy. Among the factors involved in this process, essential trace elements such as zinc and selenium stand out, as they are related to cardiovascular health and may help mitigate the harmful changes resulting from AMI. The objective of this protocol is to detail the development of two systematic reviews to gather scientific evidence on the relationship between zinc and selenium and the prognosis following AMI. This protocol was developed in accordance with the Preferred Reporting Items for Systematic Review and Meta-Analysis Protocols (PRISMA-P) guideline and registered in the International Prospective Register of Systematic Reviews (PROSPERO) under the code CRD42024574424. Search strategies will be conducted using a combination of controlled and uncontrolled terms combined with Boolean operators, and the following databases will be used: MEDLINE/PubMed, EMBASE, LILACS, Scopus, Web of Science, Trip database, and World Wide Science. Cohort studies that evaluated zinc and selenium in the prognosis after AMI will be included. Two trained researchers will independently select articles, extract data, and assess the risk of bias and the quality of the evidence. A narrative synthesis will be performed, and the main findings will be presented in tables. If possible, a meta-analysis will also be conducted.

## Introduction

Cardiovascular diseases (CVD) are the leading cause of death worldwide, accounting for approximately 32% of all deaths in 2019, according to WHO data. These conditions pose an emerging challenge to healthcare systems. Modifiable risk factors, such as diet, smoking, and physical inactivity, combined with non-modifiable factors like age, sex, and genetic predisposition, play a central role in the increasing incidence of these diseases [1,2].

Among CVD, acute myocardial infarction (AMI) stands out as one of the most common, severe, and fatal events. It is characterized by the obstruction of coronary arteries, which blocks blood flow and causes the death of millions of cardiomyocytes. This leads to an immune response and the recruitment of inflammatory cells to the infarcted area [3].

As a result of this ischemic condition, adaptive responses are triggered, such as cardiac remodeling, a compensatory and adaptive process that can cause significant morphological and functional changes in the myocardium. This includes the activation of neurohormonal reactions, mitochondrial dysfunction, oxidative stress, and inflammation, potentially progressing to adverse pathological remodeling. This progression is often associated with the development of Left Ventricular Systolic Dysfunction (LVSD), heart failure (HF), prolonged hospitalization, and increased mortality [3,4]. Among the factors involved in these pathophysiological processes, which contribute to antioxidant defense and regulate the inflammatory and healing processes, some essential trace elements, such as zinc and selenium, stand out as fundamental in mitigating harmful alterations resulting from the infarction [5,6].

Essential trace elements play a significant role in various physiological mechanisms, with selenium and zinc standing out as enzymatic cofactors that help maintain mitochondrial activity against reactive oxygen species, attenuate inflammatory responses, and regulate gene expression through transcription factors [7,8]. Inadequate selenium concentrations intensify the transformation of monocytes into foam cells, increase the likelihood of endothelial cell apoptosis, and consequently lead to greater instability of atherosclerotic plaques [7]. Similarly, zinc deficiency has been associated with structural modifications in endothelial cells and atherosclerosis [7].

Considering that inflammation and oxidative stress, integral to the complex pathophysiology of CVDs, particularly AMI, are linked to the progression of cardiac tissue hypertrophy and the transition of cardiac remodeling to HF, it has been observed that low concentrations of these trace elements are associated with increased inflammation and oxidation, heightened atherogenic processes, and a higher risk of coronary artery disease and HF [7,9,10].

However, there is no consolidated scientific evidence on the association between blood concentrations of zinc and selenium in the post-AMI period and how these trace elements may affect the prognosis of individuals who have experienced a myocardial infarction. Some systematic reviews have analyzed the difference in blood selenium concentrations between individuals with and without CVD, including AMI [11], as well as blood zinc concentrations and their possible association with the risk of developing coronary artery disease (CAD) [12].

The systematic assessment of the status of essential trace elements such as zinc and selenium in the post-AMI context will enable a rigorous analysis of the potential influence of inadequate levels compared to adequate levels on the prognosis of individuals who have experienced myocardial infarction. By comparing the status of these trace elements, we aim to identify possible associations with patient outcomes, providing a basis for more precise nutritional management strategies. Therefore, the objective of this protocol is to detail the development of two systematic reviews that will be conducted independently as well as meta-analyses for each review that will investigate the relationship between these two essential trace elements (zinc and selenium) and post-AMI prognosis. The review seeks to answer two research questions:

1. What is the association between low blood zinc concentration levels and the prognosis of patients who have experienced AMI?
2. What is the association between low blood selenium concentration levels and the prognosis of patients who have experienced AMI?

## Methodology

### Protocol registration

This protocol was developed following the Preferred Reporting Items for Systematic Review and Meta-Analysis Protocols (PRISMA-P) guideline (S1 Appendix) [13] and has been registered in the International Prospective Register of Systematic Reviews (PROSPERO) under the registration number CRD42024574424, available at: https://www.crd.york.ac.uk/prospero/display_record.php?ID=CRD42024574424.

### Review question

Two structured questions were developed based on the PECOS acronym (P, Population; E, Exposure; C, Comparison; O, Outcomes; S, Study type) (Table 1 and Table 2):

**Table 1:** Research question structure (What is the association between low blood zinc concentration levels and the prognosis of patients who have experienced acute myocardial infarction?) based on the PECOS strategy for the systematic review.

Description	Abbreviation	Elements
Population	P	Individuals who have experienced acute myocardial infarction
Exposure	E	Low blood zinc concentration levels
Comparison	C	Adequate zinc in blood concentration
Outcomes	O	Mortality, hospitalizations, heart failure
Study Type	S	Cohort studies

PECOS (P, Population; E, Exposure; C, Comparison; O, Outcomes; S, Study Type).

**Table 2:** Research question structure (What is the association between low blood selenium concentration levels and the prognosis of patients who have experienced acute myocardial infarction?) based on the PECOS strategy for the systematic review.

Description	Abbreviation	Elements
Population	P	Individuals who have experienced acute myocardial infarction
Exposure	E	Low blood selenium concentration levels
Comparison	C	Adequate selenium in blood concentration
Outcomes	O	Mortality, hospitalizations, heart failure
Study Type	S	Cohort studies

PECOS (P, Population; E, Exposure; C, Comparison; O, Outcomes; S, Study Type).

- What is the association between low blood zinc concentration levels and the prognosis of patients who have experienced AMI?
- What is the association between low blood selenium concentration levels and the prognosis of patients who have experienced AMI?

### Eligibility criteria

#### Participants/Population.

Studies including individuals who have experienced AMI will be included. Studies involving individuals with pre-existing cardiovascular conditions, such as congenital heart disease, dilated cardiomyopathy, chronic atrial fibrillation, HF, Chagas disease, those who have undergone heart transplantation, individuals diagnosed with chronic kidney disease or cancer, as well as pregnant and lactating women, will be excluded.

#### Exposure(s).

Studies that evaluated the deficiency of essential trace elements (zinc and selenium) in the post-AMI period through plasma, erythrocyte, or serum biomarkers will be included, whenever the number of publications allows, sensitivity analyses stratified by biological matrix will be conducted in order to assess the consistency of the findings. Studies that used nail or hair as biomarkers or assessed toxicity will be excluded.

#### Comparator(s)/Control.

Studies comparing individuals with adequate zinc and selenium in blood concentration post-AMI as controls will be included. Studies without control groups will be excluded.

#### Primary outcomes.

Studies evaluating outcomes such as mortality, hospitalization, and HF as primary outcomes will be included. Other clinical outcomes will be considered secondary outcomes. Studies that do not assess patient prognosis will be excluded.

#### Study type.

Observational cohort studies will be included, in order to evaluate the association with clinical outcomes. Other types of studies will be excluded.

### Information sources and literature search

A comprehensive search will be conducted without time or language restrictions, using controlled terms (MeSH, Entry terms, DeCS, EMTREE, or non-indexed terms) and Boolean operators (AND or OR) in the following electronic bibliographic databases: MEDLINE/PubMed, EMBASE, LILACS, Scopus, Web of Science, Trip database, and World Wide Science. The search strategy is presented in Tables 3 and 4. Adjustments to the search strategies may be made based on the specific characteristics of each database, and additional terms may be included or modified. To validate the search strategies, two librarians from the Federal University of Rio Grande do Norte were consulted. Additionally, a manual search of references in included studies will be performed to identify potentially eligible studies not retrieved from the databases.

**Table 3:** Search strategy in the PubMed/MEDLINE database to retrieve articles addressing the systematic review question: What is the association between low blood zinc concentration levels and the prognosis of patients who have experienced acute myocardial infarction.

Database	Search strategies
MEDLINE/PubMed	(“Inferior Wall Myocardial Infarction” OR “Anterior Wall Myocardial Infarction” OR “Myocardial Infarction” OR “Heart Rupture, Post-Infarction” OR “Non-ST Elevated Myocardial Infarction” OR “ST Elevation Myocardial Infarction” OR “MINOCA” OR “Inferior Myocardial Infarction” OR “Diaphragmatic Myocardial Infarction” OR “Acute Inferior Myocardial Infarction” OR “Anteroseptal Myocardial Infarction” OR “Anterolateral Myocardial Infarction” OR “Acute Anterior Wall Myocardial Infarction” OR “Heart Attack” OR “Myocardial Infarct” OR “Cardiovascular Stroke” OR “Post-Infarction Cardiac Rupture” OR “Post-Infarction Heart Rupture” OR “Post Infarction Heart Rupture” OR “Non ST Elevated Myocardial Infarction” OR “NSTEMI” OR “Non-ST-Elevation Myocardial Infarction” OR “Non ST Elevation Myocardial Infarction” OR “STEMI” OR “ST Segment Elevation Myocardial Infarction” OR “ST Elevated Myocardial Infarction” OR “Myocardial Infarction and Non-obstructed Coronary Arteries” OR “Myocardial Infarction and Non obstructed Coronary Arteries” OR “Myocardial Infarction and Nonobstructed Coronary Arteries” OR “Myocardial Infarction with Non-obstructive Coronary Arteries” OR “Myocardial Infarction with Non obstructive Coronary Arteries” OR “Myocardial Infarction with Nonobstructive Coronary Arteries”) AND (“Trace Elements” OR Zinc OR “Trace Element” OR Biometal OR “Trace Mineral” OR metallomics)

**Table 4:** Search strategy in the PubMed/MEDLINE database to retrieve articles addressing the systematic review question: What is the association between low blood selenium concentration levels and the prognosis of patients who have experienced acute myocardial infarction.

Database	Search strategies
MEDLINE/PubMed	(“Inferior Wall Myocardial Infarction” OR “Anterior Wall Myocardial Infarction” OR “Myocardial Infarction” OR “Heart Rupture, Post-Infarction” OR “Non-ST Elevated Myocardial Infarction” OR “ST Elevation Myocardial Infarction” OR “MINOCA” OR “Inferior Myocardial Infarction” OR “Diaphragmatic Myocardial Infarction” OR “Acute Inferior Myocardial Infarction” OR “Anteroseptal Myocardial Infarction” OR “Anterolateral Myocardial Infarction” OR “Acute Anterior Wall Myocardial Infarction” OR “Heart Attack” OR “Myocardial Infarct” OR “Cardiovascular Stroke” OR “Post-Infarction Cardiac Rupture” OR “Post-Infarction Heart Rupture” OR “Post Infarction Heart Rupture” OR “Non ST Elevated Myocardial Infarction” OR “NSTEMI” OR “Non-ST-Elevation Myocardial Infarction” OR “Non ST Elevation Myocardial Infarction” OR “STEMI” OR “ST Segment Elevation Myocardial Infarction” OR “ST Elevated Myocardial Infarction” OR “Myocardial Infarction and Non-obstructed Coronary Arteries” OR “Myocardial Infarction and Non obstructed Coronary Arteries” OR “Myocardial Infarction and Nonobstructed Coronary Arteries” OR “Myocardial Infarction with Non-obstructive Coronary Arteries” OR “Myocardial Infarction with Non obstructive Coronary Arteries” OR “Myocardial Infarction with Nonobstructive Coronary Arteries”) AND (“Trace Elements” OR Selenium OR “Trace Element” OR Biometal OR “Trace Mineral” OR “Selenium-80” OR “Selenium 80” OR metallomics)

### Study selection

**Fig 1:**
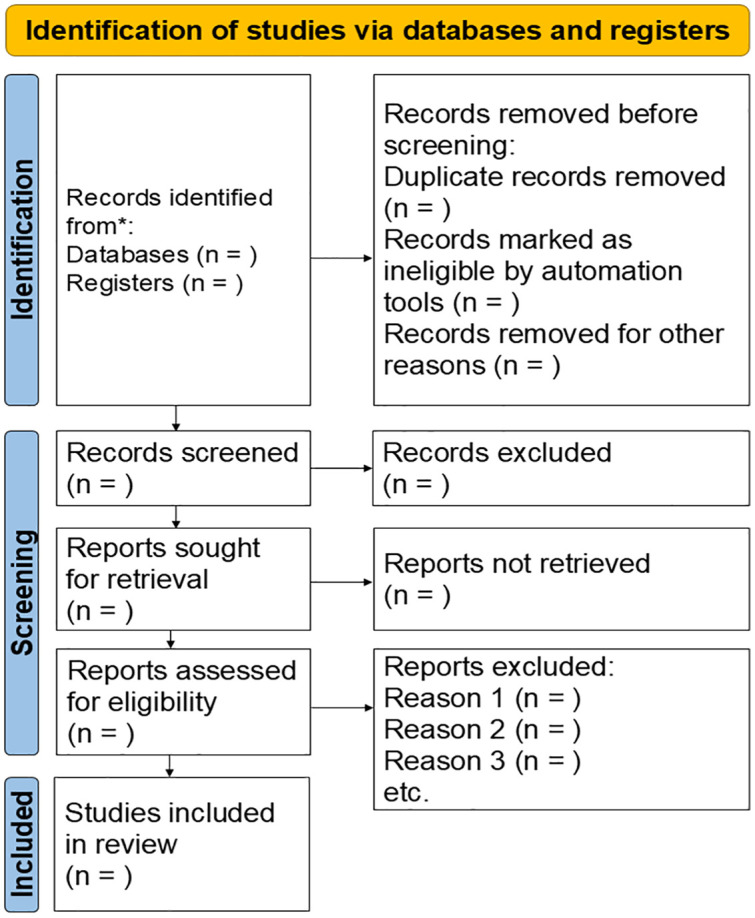
Flowchart for article selection based on the Preferred Reporting Items for Systematic Reviews and Meta-Analyses (PRISMA) [15].

After the search, all articles will be imported into the Rayyan application (version 0.1.0) [14]. The migration of articles to this platform will facilitate the removal of duplicate studies (based on inclusion criteria). The studies will be independently reviewed by two researchers. Initially, titles and abstracts will be screened. Subsequently, studies considered eligible will be read in full. A third reviewer will resolve any disagreements. The steps for study selection, as well as the reasons for study exclusion, will be recorded and presented according to the PRISMA flowchart (Fig 1) [15]. For studies not available in full text, up to three contact attempts will be made via email or through ResearchGate to reach the corresponding authors.

### Data extraction

The literature search will be updated prior to the data extraction stage in order to include studies published after the protocol registration. The following characteristics will be extracted from the selected articles: authors, country, year of publication, study type, AMI classification (according to the criteria used by the studies) sample size, population characteristics, biomarkers evaluated (types of matrices used like serum, plasma, or erythrocyte concentrations, the sensitivity analyses stratified by matrix will be conducted whenever the number of publications allows), blood concentration of trace elements, classification of trace element in blood concentration (according to values used in studies that generally classify as lower concentrations those below the median), AMI definitions (considered according to what is described in each included study, respecting the criteria established by the original authors) prognosis (death, hospitalization, and HF as primary outcomes and other prognoses as secondary outcomes), statistical methods used for analysis, risk estimates, and confidence intervals. These data will be presented in a predefined table using Microsoft Excel®. For studies with missing data, the corresponding authors will be contacted up to three times via email or ResearchGat.

### Quality assessment and risk of bias

The quality of evidence and risk of bias will be assessed independently by two researchers. The Risk Of Bias In Non-randomized Studies of Exposure (ROBINS-E) will be used to evaluate the methodological quality and risk of bias of the included studies [16]. To assess the quality of the evidence for each type of outcome in the included studies, the GRADE tool will be applied [17].

### Data synthesis strategy

The data will be summarized using a narrative approach, and the characteristics of the included studies will be described in tables. The review will be structured around the zinc and selenium in blood concentration associated with post-AMI infarction prognosis. Summaries of the results of the included studies will be provided.

If feasible, a meta-analysis for each systematic review will be performed using statistical R software (R Core Team, version 4.5.1). To assess the heterogeneity of the included studies, the Chi-squared test (X²) will be used for each outcome, with a significance level of 0.05. The I² statistic will be applied to measure the proportion of total variation across study results, with heterogeneity considered low at up to 25%, moderate between 25–50%, and high above 50%.

If possible, publication bias will be assessed using funnel plots. Funnel plot asymmetry will be evaluated using Egger’s test and Begg’s test. Where relevant, subgroup analyses may be conducted by sex or age subgroup. Considering the expected clinical and methodological heterogeneity among the studies, the quantitative synthesis will preferably be conducted using a random-effects model, which is more appropriate for integrating results from studies that are not identical but address similar research questions. The fixed-effect model will be considered only in sensitivity analyses, when there is low heterogeneity and a methodological justification for its application.

### Ethics and dissemination

This work is a study protocol and will be based on scientific literature published in different databases, dispensing with ethical approval. The results of the systematic review and meta-analysis will be published in a peer-reviewed publication and any necessary protocol changes will be recorded in the manuscript.

## Discussion

This systematic review protocol aims to systematize the search for scientific evidence to identify whether there is a relationship between zinc and selenium in blood concentration and the prognosis of individuals who suffered AMI. Only cohort studies will be included, as they allow the follow-up of individuals over time, making it possible to observe the temporal relationship between exposure (zinc and selenium levels) and the risk of developing clinical outcomes.

Trace elements are chemical components naturally found in various organisms (human, animal, plant) and even in the soil. They are involved in processes such as proper growth, development, maintenance, and health recovery [18,19]. Additionally, they act as cofactors for enzymes, stabilize the three-dimensional structures of proteins, and exhibit anti-inflammatory and antioxidant properties [19,20]. Trace elements can be classified based on their importance in physiological and biochemical processes as either essential such as selenium, zinc, copper, and manganese, and non-essential/toxic such as aluminum, cadmium, and lead [20].

The study of trace elements and their roles within cells and tissues falls under a scientific field known as metallomics. This area integrates the analysis of these elements in biological systems and has been increasingly explored in research [21]. To understand the relationship between metals and their biological and physiological functions, quantification techniques are employed, primarily using bodily fluids such as serum and plasma [21].

In the context of cardiovascular diseases (CVD)—one of the leading causes of morbidity and mortality worldwide—the study of metallomics appears promising as a strategy for discovering potential biomarkers that could aid in improving prognosis [21,22]. Essential trace elements like selenium and zinc play significant roles in cardiovascular health by modulating hydrogen and lipid hydroperoxides, maintaining endothelial cell integrity and immune system function, and acting as cofactors for enzymes like superoxide dismutase (SOD), that are crucial for heart health and proper vascular function, among other roles [12,23,24].

The involvement of trace elements in the specific context of AMI remains underexplored. Lim et al. [25] evaluated the status of essential and toxic trace elements in individuals who suffered AMI to prospect them as potential biomarkers for AMI prediction and monitoring. Using a high-performance model based on a random forest classification approach, which combined metallomic and traditional cardiovascular risk variables to predict AMI, they reported an area under the curve (AUC) of 0.942 and a high prediction accuracy of 87%. Among the top 10 most important variables, 3 were traditional cardiovascular risk factors (e.g., cholesterol and white blood cell count), while the remaining 7 were metallomic characteristics, demonstrating their potential as sensitive biomarkers for AMI prediction.

The significant issue with AMI lies in its post-ischemic complications. One such consequence is cardiac remodeling—a compensatory, adaptive, and functional process in response to injury, which may progress with changes compromising cardiac function and leading to HF [3,26]. AMI is one of the primary causes of HF, and a large proportion of patients who develop HF after AMI die within 7.6 years, underscoring the importance of monitoring individuals who suffered AMI [26].

Studies suggest that a deficiency in essential trace elements may affect ventricular function, functional capacity, and prognosis in HF [27–29]. Therefore, it is crucial to investigate the role of essential trace elements in the post-AMI context to assess their potential as risk or protective factors and even as more specific biomarkers.

The systematic review to be developed may help the scientific community address current knowledge gaps, such as determining the blood concentration of zinc and selenium necessary to prevent unfavorable outcomes, supporting future studies like experimental research in animal models and cells, as well as clinical trials to evaluate supplementation effects and identify effective dosages. It could also prospect new biomarkers and contribute to clinical protocols for nutritional care, providing evidence-based recommendations to enhance cardiovascular health and quality of life for individuals who have suffered AMI.

## Supporting information

S1 Appendix Preferred Report Items for Systematic Reviews and Meta-analyses (PRISMA-P) checklist. (PDF)
